# Monitor unit optimization in stereotactic body radiotherapy for small peripheral non-small cell lung cancer patients

**DOI:** 10.1038/srep18453

**Published:** 2015-12-18

**Authors:** Bao-Tian Huang, Zhu Lin, Pei-Xian Lin, Jia-Yang Lu, Chuang-Zhen Chen

**Affiliations:** 1 Cancer Hospital of Shantou University Medical College, Department of Radiation Oncology, Shantou, 515031, China; 2 The Second Affiliated Hospital of Shantou University Medical College, Department of Nosocomial Infection Management, Shantou, 515041, China

## Abstract

The increasingly attractive stereotactic body radiotherapy (SBRT) treatment for stage I lung cancer is concomitant with a large amount of monitor units (MU), leading to excessive out-of-field dose and prolonged beam-on time. The study aims to reduce the MU number and shorten the beam-on time by optimizing the planning parameters. Clinically acceptable treatment plans from fourteen patients suffered from peripheral stage I non-small cell lung cancer (NSCLC) were created in the study. Priority for the upper objective of the target (PUOT), strength and Max MU setting in the MU objective function (MUOF) were adjusted respectively to investigate their effect on MU number, organs at risk (OARs) sparing and beam-on time. We found that the planning parameters influenced the MU number in a PUOT, strength and Max MU dependent manner. Combined with high priority for the UOT (HPUOT) and MUOF, the MU number was reduced from 443 ± 25 to 228 ± 22 MU/Gy without compromising the target coverage and OARs sparing. We also found beam-on time was proportional to MU number and it could be shortened from 7.9 ± 0.5 to 4.1 ± 0.4 minutes.

Retrospective studies have demonstrated that stereotactic body radiotherapy (SBRT) treatment was effective for medically inoperable early stage non-small cell lung cancer (NSCLC)^1^,^2^,^3^. It has been reported to achieve similar overall survival (OS), disease-free survival (DFS), local control (LC) and distant control (DC) as surgery while maintaining minimal toxicity^4^,^5^,^6^. The latest research in Lancet Oncology brings in the encouraging result that SBRT strategy achieves better outcome than surgery for operable stage I NSCLC^7^.

Single-fraction SBRT strategy has been widely used for lung cancer^8^,^9^,^10^ and several publications have demonstrated its safety, efficacy, and minimal toxicity for NSCLC treatment^11^,^12^. As the dose regimen was usually larger than 25 Gy per fraction in single-fraction SBRT, the excessive monitor units (MU) and prolonged beam-on time has become an issue of concern. It was reported the required MU was in the range of 2000–10000 for a fraction dose in excess of 10 Gy^13^ and the average beam-on time ranged from 5 to 6 minutes in SBRT treatment with 25 Gy per fraction^10^,^14^. The MU number and beam-on time will be more than reported when larger dose regimens such as 30 Gy or 34 Gy was used. It has been estimated that doubled MU could translate into a potential increase in the risk of secondary cancers by a factor of 1.2–8^15^,^16^ and the extended treatment time could increase the risk of tumor displacement during beam delivery^17^,^18^.

Eclipse treatment planning system (TPS) has incorporated the MU objective function (MUOF) to reduce MU number during treatment planning. To our knowledge, few researches have investigated the effect of planning parameters on MU number and beam-on time, particularly for SBRT treatment of lung cancer which always involves high dose fractionation.

Therefore, the study aims to reduce the MU number and beam-on time for SBRT treatment of lung cancer by optimizing the planning parameters in Eclipse.

## Materials and Methods

### Ethics statement

The protocol was approved by the Ethics Committee of Cancer Hospital of Shantou University Medical College. Since this is not a treatment-based study, our institutional review board waived the need for written informed consent from the participants. The methods in the study were performed in accordance with the approved guidelines and regulations.

### CT scanning and contouring of organs at risk (OARs)

During April 2013 and May 2015, fourteen patients suffering from stage I lung cancer were enrolled in this study. Basic information of the patients was listed in Table 1. Patients were positioned supine on a vacuum bag (Medtec Medical, Inc, Buffalo Grove, IL) or a thermoplastic mask (Guangzhou Klarity Medical & Equipment Co., Ltd, Guangzhou, China). Patients were subjected to a four-dimensional computed tomography (4DCT) scans with Brilliance Big Bore CT (Philips, Inc, Netherlands) under uncoached free breathing. The CT images were then transferred to Eclipse TPS (V10, Varian Medical System, Inc., Palo Alto, CA) for target volume, OARs delineation and treatment planning. The gross target volume (GTV) accounting for ten breathing phases were contoured in the pulmonary windows by a radiation oncologist specialized in lung SBRT. Ten phases of the GTV were then used to form internal target volume (ITV). To account for setup inaccuracies and potential intra-fractional tumor shift, a 0.5 cm setup margin was added to the ITV to form the planning target volume (PTV). All plans were carried out on the averaged 4DCT. OARs, including aorta, esophagus, bronchial tree, heart, spinal cord, lung and chest wall (CW) were contoured according to the RTOG 0915 report^19^.

### Treatment Planning

We prescribed 1 fraction of 25 Gy in all plans for small peripheral tumor according to previous publications^10^,^14^,^20^. Treatment planning was designed with dual partial arcs to exclude the entrance of the beam through the contralateral lung. All plans required a clockwise and a counterclockwise arc for each fraction. Collimator angles for all plans were set to 30° in one arc and 330° for another to minimize the contribution of the tongue-and-groove effect to the dose. The grouped fields were aligned to the center of the PTV. A dose-limiting structure (2 cm from PTV in any direction) was constructed to ensure a rapid dose fall-off outside the target. We used 6MV flattening filter free (FFF) beam on a TrueBeam Linac (Varian Medical Systems, Inc, Palo Alto, CA), selecting a maximum dose rate of 1400 MU/min and 114 control points to design the treatment plans. Optimization was performed with the progressive resolution optimizer (PRO) algorithm implemented in Eclipse 10.0. The objectives were adjusted to make sure the maximum dose was at least 120% of the prescription dose and centered in the GTV. Dose calculations were performed using the Anisotropic Analytic Algorithm (AAA) with a calculation grid size of 1 mm, taking into account heterogeneity correction. The plan calculated at the first time was used as a basedose plan for further optimization to compensate any underdose or “dose cloud” areas. The final dose calculation was normalized to guarantee that 95% of the PTV received the prescribed dose. All of the dose constraints, such as PTV, conformity of prescribed dose, intermediate dose spillage and critical organ dose-volume limits met the criterion of the RTOG 0915 protocol using single fraction^19^.

### Planning parameters

Priority for the upper objective of the target (PUOT) and two MU constraints (strength and Max MU) in the MUOF were adjusted to investigate their effect on MU number, target coverage, OARs sparing and beam-on time. Upper objective of the target (UOT) is the objective in the optimizer to limit the maximum dose in the target and the priority for it determines the relative importance to achieve the objective. For example, if the UOT is set to 110% of the prescribed dose and given high priority, the optimizer will ensure the target receives no more than 110% of the prescribed dose taking the first priority. The MUOF tends to keep the MU number as low as possible in the optimization process, if clinically required. When using the MUOF, we need to define the strength and the Max MU values for the objective. The strength and Max MU values can be defined between 0–100 and 0–100000, respectively.

In the study, the priority for the lower objective of the target (PLOT) was set to 100 for all the treatment plans. Relatively, PUOT was set to 60 (low priority, LPUOT), 80 (medium priority, MPUOT) and 100 (high priority, HPUOT) to investigate their effects on MU number. Strength value in the MUOF was set to 25, 50, 75 and 100, respectively. Max MU setting was set to 100%, 85%, 75%, 50% and 25%, respectively. We must emphasize that 100%, 85%, 75%, 50% and 25% Max MU setting in the MUOF equals to 100%, 85%, 75%, 50% and 25% of total MU number calculated in the LPUOT, MPUOT and MPUOT plans without the MUOF. When changing the parameters investigated, we kept other optimization parameters unchanged during the RapidArc optimization to avoid their influence on the result of treatment plans.

### Evaluation parameters

The evaluation parameters include the maximum, minimum and mean dose to the PTV. Conformity of prescribed dose (CI_100%_) was defined as the ratio of the volume of prescription isodose to the volume of PTV. Two parameters of D_2cm_ and R_50%_ were used to evaluate the intermediate dose spillage according the RTOG 0915 protocol^19^. D_2cm_ was defined as the maximum dose 2 cm away from PTV in any direction. R_50%_ was defined as the ratio of the volume of 50% prescription isodose to the volume of PTV. For the OARs, the analysis included the maximum dose to the aorta, esophagus, bronchial tree, heart and spinal cord. Meanwhile, the mean dose and a set of appropriate V_x_ values were used to assess the lung. V_x_ was the volume of the organ receiving a dose of x or more. For example, V_40_ was the volume of organ receiving a dose of 40 Gy or more. Although it was reported that V_30_ was a predictive parameter of radiation induced CW pain^21^, we have ignored the dosimetric change of CW due to the 1 × 25 Gy fraction scheme used in our study.

### Statistical analysis

All data in this study were presented as the mean plus standard deviation (mean ± SD). The Statistical Package for Social Sciences (SPSS, version 17.0, Chicago, IL) was used for statistical analysis in the present study. We used the Friedman Test to determine the difference between groups. Comparisons of the sub-group data were compared using Wilcoxon signed-rank test. Differences were considered statistically significant when *p* < 0.05.

## Results

### Effect of PUOT on MU number

**Figure 1:**
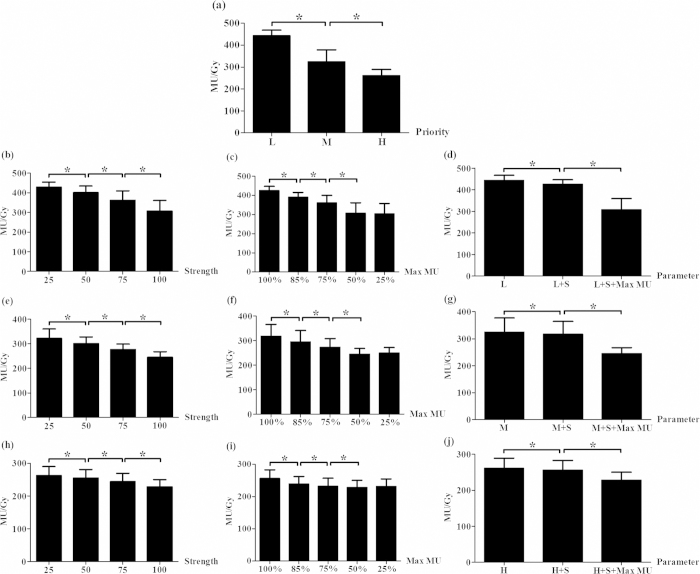
Effect of planning parameters on MU number. (**a**) impact of PUOT without MUOF; **(b,e,h) and (c,f,i)** impact of strength and Max MU setting in LPUOT, MPUOT and HPUOT plans; **(d,g,j)** MU comparison with and without the MUOF in LPUOT, MPUOT and HPUOT groups, respectively; L = LPUOT, M = MPUOT, H = HPUOT, S = strength setting, Max MU = Max MU setting. S was set to 100 and Max MU was set to 50%. ^*^indicates statistical significance (*p* < 0.05) using Wilcoxon signed-rank test.

**Figure 2:**
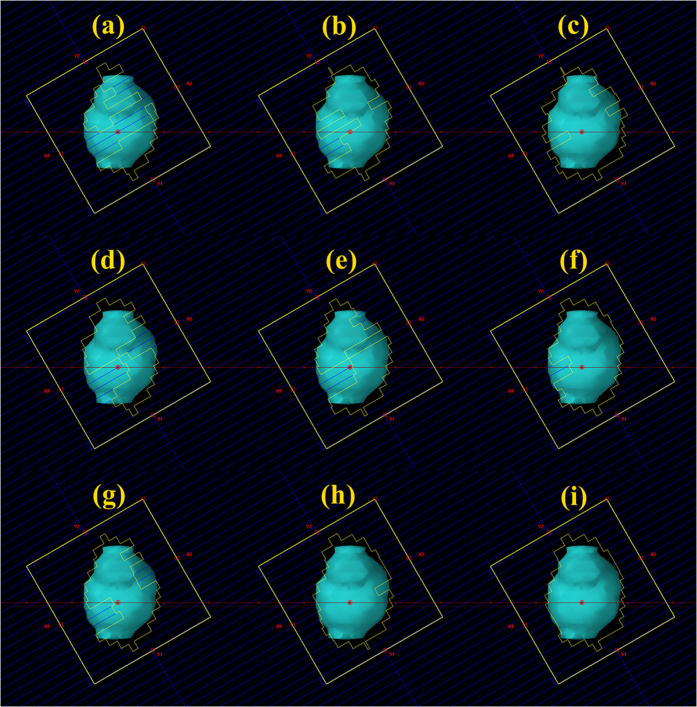
MU related MLC movement in different planning parameters. **(a–c)** LPUOT, MPUOT and HPUOT plans without MUOF; **(d–f)** LPUOT, MPUOT and HPUOT plans combined with 100 strength setting; **(g–i)** LPUOT, MPUOT and HPUOT plans combined with 100 strength and 50% Max MU setting.

We found that the planning parameters in the optimizer influenced the MU number in a PUOT, strength and max MU dependent manner. MU number decreased with the PUOT increasing. MU number was 443 ± 25, 324 ± 55 and 260 ± 28 MU/Gy for the LPUOT, MPUOT and HPUOT groups, respectively. Effect of PUOT on the MU number was exhibited in Fig. 1a. The HPUOT group also spared the heart, spinal cord and lung while maintaining comparable target coverage than the other two groups. Dosimetric comparison of PTV and OARs for the LPUOT, MPUOT and HPUOT plans without the MUOF were shown in Table 2. Reduction of MU number was associated with less multileaf collimator (MLC) movement. MU related MLC movement in LPUOT, MPUOT and HPUOT groups without the MUOF were exhibited in Fig. 2a–c.

### Effect of MUOF on MU number

The strength and Max MU settings in the MUOF also influenced the MU number. MU number was continuously reduced with the strength increasing and Max MU setting decreasing, irrespective of LPUOT, MPUOT and HPUOT groups. With the maximum strength of 100, MU number reached the minimum while maintaining comparable dose to the OARs. We also found that the MU number continued to decrease at 100%, 85%, 75% and 50% Max MU setting, but no longer to decrease at 25%. Effect of the MUOF (strength and Max MU setting) on MU number in the LPUOT, MPUOT and HPUOT groups were also displayed in Fig. 1b–j. MLC movement with the MUOF in LPUOT, MPUOT and HPUOT groups were exhibited in Fig. 2d–i. Dosimetric comparison of PTV and OARs with and without the MUOF in the LPUOT, MPUOT and HPUOT plans were shown in Table 3, 4, 5, respectively.

Combined with HPUOT and MUOF, MU number could be reduced to as low as 228 ± 22 MU/Gy (Fig. 1j) without compromising the dose to the target and OARs (Table 5).

### Effect of planning parameters (PUOT and MUOF) on beam-on time

**Figure 3:**
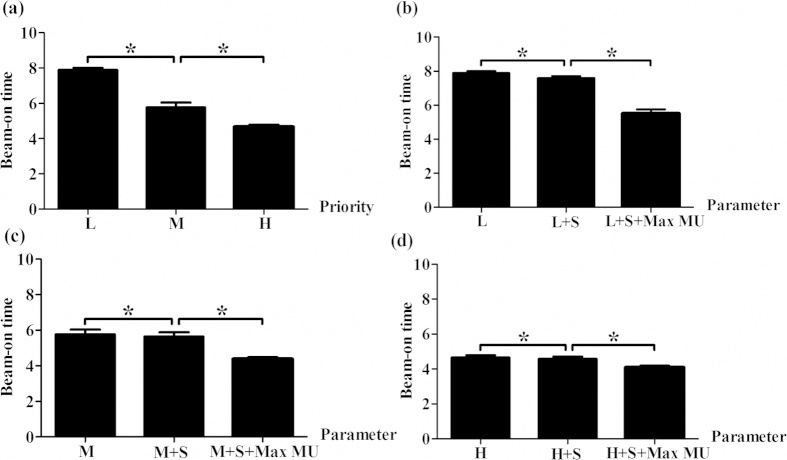
Effect of planning parameters on beam-on time. **(a)** impact of PUOT without MUOF; **(b–d)** impact of the MUOF in LPUOT, MPUOT and HPUOT groups; L = LPUOT, M = MPUOT, H = HPUOT, S = strength setting, Max MU = Max MU setting. S was set to 100 and Max MU was set to 50%. ^*^indicates statistical significance (*p* < 0.05) using Wilcoxon signed-rank test.

We also found that beam-on time was proportional to MU number and the corresponding beam-on time for the LPUOT, MPUOT and HPUOT plans were 7.9 ± 0.5, 5.9 ± 1.0 and 4.7 ± 0.5 minutes (Fig. 3a). When incorporating the MUOF, mean beam-on time was furtherly reduced to 5.5 ± 1.0, 4.4 ± 0.4 and 4.1 ± 0.4 minutes for the LPUOT, MPUOT and HPUOT plans, respectively. Effect of the MUOF (strength and Max MU) on beam-on time in the LPUOT, MPUOT and HPUOT plans was exhibited in Fig. 3b–d.

## Discussion

In this study, we have optimized the planning parameters to reduce the MU number and thus shorten the beam-on time. We found that the planning parameters influenced the MU number in a PUOT, strength and Max MU dependent manner. Combined with HPUOT, maximum strength of 100 and 50% Max MU setting in the MUOF, we reduce the MU number to as low as 228 ± 22 MU/Gy in SBRT treatment for lung cancer with 1 × 25 Gy fraction scheme. Meanwhile, the corresponding beam-on time was shortened to 4.1 ± 0.4 minutes. To the best of our knowledge, it was the first study to investigate the effect of PUOT and MUOF on the MU number for SBRT treatment of lung cancer involving high dose fraction scheme.

Clemente *et al.* had investigated the MU objective tool incorporated in the Eclipse TPS to reduce the MU number in prostate cancer patients using a conventional fractionation of 2 Gy per day^22^. They observed that the favorable combination of 100 strength and 50% Max MU in the MUOF could result in 28% reduction in the MU number whereas the deterioration in homogeneity index (HI) of the target was up to 23%. Moreover, they also found that the rectum and femoral heads dose were significantly higher in the MU-optimized group than that without incorporating the function. Our data that the combination of 100 strength and 50% Max MU setting achieved the lowest MU number was highly in accordance with their result. However, our finding that the function significantly reduced the total MU number (up to 15%) while maintaining comparable target coverage and improved OARs sparing differed from theirs. One explanation for the inconsistency is that the OARs are away from the target in peripheral lung patients but the OARs like bladder, rectum, small intestine and femoral heads are adjacent to the target in prostate cases. The anatomical characteristics in prostate cases might lead to the HI deterioration and more irradiation to the OARs when the MU number was enforced to be reduced. From the two independent researches, we speculate that the MUOF is more beneficial to cases where the target is away from the OARs, such as peripheral lung cancer patients and so on. When the target is adjacent to the OARs, MU number decreased at the cost of compromising HI of the target and OARs sparing. To our knowledge, this point has not been proposed and this study is the first to report and analyze it.

A waste of MU is worthy of attention in modern radiotherapy, particularly in intensity modulated radiotherapy (IMRT) treatment which can increase the MU number by a factor of 2 to 10 (typically 3–5) depending on the techniques and equipment used^23^. It is known and accepted that the scattered radiation administered to a patient’s body outside of the treatment volume is at first-order directly proportional to the applied MU in treatments with linear accelerators^24^. Excessive MU increases the leakage radiation and out-of-field dose, and simultaneously enhances the risk of radiation induced second malignancies^15^,^23^,^25^. The issue of excessive MU and the radiation induced second malignancies needs to be taken into consideration due to the large fractional dose and good life expectancy achieved in SBRT treatment of lung cancer. The reported single-fraction SBRT used fractional dose up to 34 Gy^9^ and the total MU number will be larger compared with the 1 × 25 scheme in our study. Additionally, it was reported that 2-year OS and LC were 70% and 91% in 3201 patients with localized stage I NSCLC from a systematic review^4^. With treatment becoming more successful and survival rates rising, lowering the risk of radiation induced secondary cancer is of particular concern in lung SBRT treatment.

MU reduction also translates into beam-on time shortening. Many publications have reported the potential of volumetric modulated arc therapy (VMAT) combined FFF beams to improve the treatment efficiency compared with conventional IMRT^13^,^26^,^27^,^28^,^29^. However, in our study, we found the treatment efficiency could be further improved by optimizing the planning parameters. Shorter treatment time generally translated into substantially superior patient stability and treatment accuracy^17^, simultaneously reduced the likelihood of intrafractional baseline shifts in tumor position^18^. For high dose SBRT treatment of lung cancer, reducing the beam-on time is of clinical significance for two reasons: *(1)* Previous research regarding the target motion as a function of treatment time found the average time needed to maintain the target motion within 1 mm of translation or 1 degrees of rotational deviation was 5.9 min for thoracic tumors^30^. In our study, the beam-on time was shortened to less than 5.5 minutes on average with the MUOF, implying a stable target motion during the delivery. *(2)* For SBRT treatment with FFF beams which provides as high as 2400 MU/min dose rate, a maximum dose of 1 Gy can be delivered within the course of 2.5 seconds^13^. Therefore, it was important to shorten the beam-on time in SBRT treatment for lung patients, particularly when the large dose fraction schemes like 1 × 25 Gy, 1 × 30 Gy or 1 × 34 Gy were used.

Reducing the MU number also means less MLC modulation is required to achieve comparable dose distribution (Fig. 2). MLC interlock is a common linac accelerator (LA) failure and reduction of MLC movement probably helps to lower the MLC workload. Moreover, it also contributes to reduce the probability of dosimetric inaccuracy induced by MLC positioning error. It was reported the average changes in D_95%_ caused by this errors was up to 8% in complex head and neck plans^31^.

Although the original goal of our research is to reduce the MU number in small peripheral NSCLC (≤3 cm) patients, we believe our experience is also useful for larger tumors (>3 cm) or other treatment sites because the tumor size doesn’t impact on the PUOT and MUOF setting. However, we need more experiments to confirm the speculation. It will be another interesting work for our future studies.

Although we have optimized the planning parameters to reduce the MU number, it does have some limitations. *(1)* The dose displayed in the study is physical dose. As SBRT treatment always involves large dose per fraction, it is necessary to convert the physical dose to biologically equivalent dose (BED) to account for the radiobiological effect. However, as the dose difference between the groups is so small that we have ignored the contribution of it. *(2)* Although the combination of HPUOT and MUOF is capable of reducing the MU number, it slightly prolongs the planning time by about 10–12 minutes because we need to know the total MU number calculated in HPUOT plan when using 50% Max MU setting. However, we found the planning parameters in study didn’t have any influence on the optimization time during each optimization.

## Conclusions

The planning parameters in the optimizer influence the MU number in a PUOT, strength and Max MU dependent manner. Combined with HPUOT and MUOF, the MU number can be reduced to 228 ± 22 MU/Gy while maintaining comparable or improved OAR sparing in SBRT treatment for lung cancer.

## Additional Information

**How to cite this article**: Huang, B.-T. *et al.* Monitor unit optimization in stereotactic body radiotherapy for small peripheral non-small cell lung cancer patients. *Sci. Rep.* **5**, 18453; doi: 10.1038/srep18453 (2015).

## Figures and Tables

**Table 1:** Characteristics of fourteen small peripheral NSCLC patients.

Patient	Gender	Age	Stage^*^	GTV (cc)	PTV (cc)	Location
1	F	71	T1	7.0	28.7	LLL
2	M	71	T1	3.1	16.3	RML
3	M	68	T1	3.6	27.6	RML
4	F	72	T1	5.4	31.3	LLL
5	M	64	T1	3.3	23.0	RML
6	M	68	T1	4.2	22.4	RML
7	M	70	T1	9.8	40.8	RLL
8	M	62	T1	9.7	63.5	RUL
9	F	63	T1	4.5	39.9	RML
10	F	70	T1	10.0	39.5	RUL
11	F	55	T1	2.0	20.2	LUL
12	M	73	T1	3.5	18.3	RML
13	F	59	T1	4.0	32.9	RML
14	M	62	T1	3.4	20.0	LLL

*Abbreviations:* GTV = gross target volume; PTV = planning target volume; LLL = left lower lobe; RML = right middle lobe; RLL = right lower lobe; RUL = right upper lobe; LUL = left upper lobe. ^*^According to American Joint Committee on Cancer (AJCC), 7^th^ edition.

**Table 2:** Dosimetric comparison of target and OARs in LPUOT, MPUOT and HPUOT plans without MUOF.

Metrics	Unit	Low	Medium	High	*p*
PTV D_min_	%	92.9 ± 0.6	93.4 ± 1.0	93.5 ± 1.0	0.257
PTV D_max_	%	123.9 ± 1.3	123.4 ± 1.3	123.2 ± 1.0	0.089
PTV D_mean_	%	108.6 ± 1.4	108.7 ± 1.3	108.7 ± 1.2	0.241
Aorta D_max_	Gy	8.3 ± 5.3	8.1 ± 5.5	8.1 ± 5.5	0.931
Esophagus D_max_	Gy	5.4 ± 1.7	5.2 ± 1.3	5.1 ± 1.4	0.395
Bronchial tree D_max_	Gy	7.3 ± 5.4	7.1 ± 5.4	7.2 ± 5.3	0.395
Heart D_max_	Gy	7.0 ± 3.7	7.1 ± 3.7	6.7 ± 3.7	0.003
Cord D_max_	Gy	5.4 ± 2.2	5.0 ± 2.1	4.7 ± 2.0	0.024
Lung V_5_	%	9.6 ± 4.1	9.5 ± 4.2	9.5 ± 4.2	0.038
Lung V_10_	%	4.6 ± 2.4	4.5 ± 2.4	4.5 ± 2.4	0.012
Lung V_20_	%	1.5 ± 1.0	1.5 ± 1.0	1.5 ± 1.0	0.565
Lung D_mean_	Gy	1.6 ± 0.6	1.6 ± 0.6	1.6 ± 0.6	0.000
D_2cm_	%	49.2 ± 3.6	48.6 ± 4.0	48.7 ± 3.4	0.191
CI_100%_	–	1.00 ± 0.04	0.97 ± 0.02	0.97 ± 0.02	0.000
R_50%_	–	4.20 ± 0.37	4.14 ± 0.34	4.13 ± 0.32	0.011

*Abbreviations:* PTV = planning target volume; D_min_ = minimum dose; D_max_ = maximum dose; D_mean_ = mean dose; D_2cm_ = maximum dose 2 cm from PTV in any direction; CI_100%_ = conformity of the prescribed dose; R_50%_ = ratio of the volume of the 50% prescription isodose to the volume of PTV; Low, Medium and High = LPUOT, MPUOT and HPUOT. *p* < 0.05 stands for statistically significant between groups with Friedman Test.

**Table 3:** Dosimetric comparison of target and OARs with and without MUOF in the LPUOT plans.

Metrics	Unit	Low	Low+S	Low+S+Max	*p*
PTV D_min_	%	93.5 ± 1.0	93.4 ± 0.7	93.3 ± 0.8	0.232
PTV D_max_	%	123.3 ± 1.0	123.1 ± 1.6	123.5 ± 0.9	0.927
PTV D_mean_	%	108.8 ± 1.3	108.8 ± 1.2	108.7 ± 1.1	0.353
Aorta D_max_	Gy	7.9 ± 5.5	8.0 ± 5.5	7.8 ± 5.4	0.223
Esophagus D_max_	Gy	5.1 ± 1.4	5.1 ± 1.5	4.9 ± 1.4	0.004
Bronchial tree D_max_	Gy	7.2 ± 5.3	7.1 ± 5.2	7.0 ± 5.1	0.751
Heart D_max_	Gy	6.7 ± 3.7	6.8 ± 3.7	6.8 ± 3.8	0.410
Cord D_max_	Gy	4.7 ± 2.0	4.8 ± 1.9	4.9 ± 2.0	0.607
Lung V_5_	%	9.5 ± 4.2	9.5 ± 4.2	9.5 ± 4.2	0.505
Lung V_10_	%	4.5 ± 2.4	4.6 ± 2.4	4.5 ± 2.4	0.423
Lung V_20_	%	1.5 ± 1.0	1.5 ± 1.0	1.5 ± 1.0	0.846
Lung D_mean_	Gy	1.6 ± 0.6	1.6 ± 0.6	1.6 ± 0.6	0.629
D_2cm_	%	48.7 ± 3.4	49.0 ± 3.4	48.7 ± 3.0	0.569
CI_100%_	—	0.97 ± 0.02	0.97 ± 0.02	0.97 ± 0.02	0.657
CI_50%_	—	4.14 ± 0.32	4.15 ± 0.31	4.15 ± 0.31	0.423

*Abbreviations:* PTV = planning target volume; D_min_ = minimum dose; D_max_ = maximum dose; D_mean_ = mean dose; D_2cm_ = maximum dose 2cm from PTV in any direction; CI_100%_ = conformity of the prescribed dose; R_50%_ = ratio of the volume of the 50% prescription isodose to the volume of PTV; Low = LPUOT; Low+S = LPUOT combined with 100 strength setting; Low+S+Max = LPUOT combined with 100 strength and 50% Max MU setting. *p* < 0.05 indicates statistically significant between groups with Friedman Test.

**Table 4:** Dosimetric comparison of target and OARs with and without MUOF in the MPUOT plans.

Metrics	Unit	Medium	Medium+S	Medium+S+Max	*p*
PTV D_min_	%	93.5 ± 1.0	93.4 ± 0.8	93.4 ± 0.8	0.744
PTV D_max_	%	123.2 ± 0.9	123.6 ± 1.1	122.9 ± 1.5	0.066
PTV D_mean_	%	108.9 ± 1.1	108.8 ± 1.2	108.8 ± 1.3	0.779
Aorta D_max_	Gy	7.8 ± 5.6	7.9 ± 5.5	8.0 ± 5.4	0.062
Esophagus D_max_	Gy	5.1 ± 1.4	5.1 ± 1.5	4.8 ± 1.4	0.002
Bronchial tree D_max_	Gy	7.2 ± 5.3	7.1 ± 5.2	7.0 ± 5.2	0.410
Heart D_max_	Gy	6.8 ± 3.7	6.8 ± 3.8	6.9 ± 3.8	0.931
Cord D_max_	Gy	4.8 ± 2.0	4.9 ± 2.0	4.8 ± 1.8	0.607
Lung V_5_	%	9.5 ± 4.2	9.5 ± 4.2	9.5 ± 4.2	0.281
Lung V_10_	%	4.6 ± 2.4	4.5 ± 2.4	4.5 ± 2.4	0.558
Lung V_20_	%	1.5 ± 1.0	1.5 ± 1.0	1.5 ± 1.0	0.846
Lung D_mean_	Gy	1.6 ± 0.6	1.6 ± 0.6	1.6 ± 0.6	0.472
D_2cm_	%	48.8 ± 2.9	49.0 ± 3.4	48.7 ± 3.4	0.404
CI_100%_	–	0.97 ± 0.01	0.97 ± 0.01	0.97 ± 0.02	0.397
CI_50%_	–	4.14 ± 0.32	4.16 ± 0.32	4.15 ± 0.31	0.212

*Abbreviations:* PTV = planning target volume; D_min_ = minimum dose; D_max_ = maximum dose; D_mean_ = mean dose; D_2cm_ = maximum dose 2 cm from PTV in any direction; CI_100%_ = conformity of the prescribed dose; R_50%_ = ratio of the volume of the 50% prescription isodose to the volume of PTV; Medium = MPUOT; Medium+S = MPUOT combined with 100 strength setting; Medium+S+Max = MPUOT combined with 100 strength and 50% Max MU setting. *p* < 0.05 indicates statistically significant between groups with Friedman Test.

**Table 5:** Dosimetric comparison of target and OARs with and without MUOF in the HPUOT plans.

Metrics	Unit	High	High+S	High+S+Max	*p*
PTV D_min_	%	93.6 ± 1.0	93.4 ± 0.7	93.2 ± 0.9	0.123
PTV D_max_	%	123.2 ± 1.0	123.6 ± 1.1	122.9 ± 1.5	0.023
PTV D_mean_	%	108.8 ± 1.2	108.8 ± 1.2	108.7 ± 1.2	0.563
Aorta D_max_	Gy	8.1 ± 5.5	7.9 ± 5.5	7.8 ± 5.4	0.135
Esophagus D_max_	Gy	5.1 ± 1.4	5.1 ± 1.5	4.7 ± 1.4	0.005
Bronchial tree D_max_	Gy	7.2 ± 5.3	7.1 ± 5.2	7.0 ± 5.2	0.931
Heart D_max_	Gy	6.7 ± 3.7	6.8 ± 3.7	6.8 ± 3.9	0.708
Cord D_max_	Gy	4.7 ± 2.0	4.9 ± 2.0	4.8 ± 1.9	0.807
Lung V_5_	%	9.5 ± 4.2	9.5 ± 4.2	9.5 ± 4.2	0.853
Lung V_10_	%	4.5 ± 2.4	4.5 ± 2.5	4.5 ± 2.4	0.814
Lung V_20_	%	1.5 ± 1.0	1.5 ± 1.0	1.5 ± 1.0	0.100
Lung D_mean_	Gy	1.6 ± 0.6	1.6 ± 0.6	1.6 ± 0.6	0.929
D_2cm_	%	48.6 ± 3.3	48.9 ± 3.4	48.7 ± 3.0	0.931
CI_100%_	–	0.97 ± 0.02	0.97 ± 0.01	0.97 ± 0.02	0.368
CI_50%_	–	4.13 ± 0.32	4.14 ± 0.31	4.15 ± 0.31	0.074

*Abbreviations:* PTV = planning target volume; D_min_ = minimum dose; D_max_ = maximum dose; D_mean_ = mean dose; D_2cm_ = maximum dose 2 cm from PTV in any direction; CI_100%_ = conformity of the prescribed dose; R_50%_ = ratio of the volume of the 50% prescription isodose to the volume of PTV; High = HPUOT; High+S = HPUOT combined with 100 strength setting; High+S+Max = HPUOT combined with 100 strength and 50% Max MU setting. *p* < 0.05 indicates statistically significant between groups with Friedman Test.
